# The effectiveness of a psycho-education intervention on mental health literacy in communities affected by the COVID-19 pandemic—a cluster randomized trial of 24 villages in central Uganda—a research protocol

**DOI:** 10.1186/s13063-021-05391-6

**Published:** 2021-07-13

**Authors:** Dickens Akena, Ronald Kiguba, Wilson W. Muhwezi, Brendan Kwesiga, Gwendolyne Kigozi, Noeline Nakasujja, Hafsa Lukwata

**Affiliations:** 1 Department of Psychiatry, Makerere University College of Health Sciences, Kampala, Uganda; 2 Department of Pharmacology, Makerere University College of Health Sciences, Kampala, Uganda; 3 Health Systems Strengthening Cluster, World Health Organization, Kenya Country Office, Nairobi, Kenya; 4 Grants office, Makerere University College of Health Sciences, Kampala, Uganda; 5 Department of Mental Health, Ministry of Health of Uganda, Kampala, Uganda

**Keywords:** Mental health literacy, Common mental disorders, Sub-Saharan Africa

## Abstract

**Background:**

Literature shows a high prevalence of psychological distress (PD) as well as common mental disorders (CMD) such as major depressive disorders (MDD), generalized anxiety disorders (GAD), post-traumatic stress disorders (PTSD), and substance misuse disorders (SUD) among people exposed to disasters and pandemics like the COVID-19. Moreover, CMD are associated with increased mortality (mainly through suicide) and morbidity (loss of productivity). A number of countries have made deliberate efforts to identify and manage CMD in light of COVID-19. However, low levels of mental health literacy (MHL) manifested by the individual’s unawareness of CMD symptoms, limited human and mental health infrastructure resources, and high levels of mental illness stigma (MIS) are barriers to integration of mental health care in general health care during pandemics and epidemics such as the COVID-19.

**Objectives:**

For the proposed study, we will determine effectiveness of a psycho-education intervention delivered by village health team (VHT) members.

**Methods:**

We will employ a cluster randomized trial design in 24 villages in central Uganda. We will collect baseline data to and document the prevalence of MHL, PD, MDD, PTSD, GAD, and SUD. We will distribute information education and communication materials (IEC) aimed at improving MHL to 420 adult individuals in the intervention arm (*n* = 12 villages). In the control arm (*n* = 12 villages), VHTs will distribute ministry of health COVID-19 information leaflets to 420 participants. Within 7 days of distributing the materials, research assistants will conduct a follow-up interview and assess for the same parameters (MHL, PD, MDD, PTSD, GAD, and SUD). We will use an intention to treat analysis to estimate the effectiveness of the psycho-education intervention.

**Discussion:**

Findings from this research will guide policy and practice regarding the integration of mental health services in the community in the context of epidemic preparedness and response.

**Trial registration:**

ClinicalTrials.govNCT04616989. Registered on 05 November 2020

## Background

During disasters and pandemics such as the COVID-19, up to 10% of individuals in the community may suffer from moderate to severe psychological distress (PD) [1–3]. In the majority of individuals, PD is transient and abates by itself [4]. However, in some individuals, severe PD resulting from exposure to disasters may trigger the onset of common mental disorders (CMD) such as major depressive disorders (MDD), generalized anxiety disorders (GAD), post-traumatic stress disorders (PTSD), and substance misuse disorders (SUD) [5–7].

Moreover, literature shows that CMD predicts poor outcomes including high mortality (mainly through suicide) [8–10] and morbidity (low productivity and poor quality of life) [11, 12]. Due to the high prevalence of CMD among individuals exposed to disasters and the adverse events that result from it, a number of scholars have recommended that CMD are identified and adequately treated using psychological or pharmacological therapies [13–16]. However, despite the huge burden and negative consequences, CMD are rarely identified and inadequately treated in low resourced sub-Saharan Africa (SSA). A number of barriers may be responsible for the poor identification of CMDs.

First, there is a severe shortage of specialized mental health care workers in SSA—with just a psychiatrist per million persons in low resourced settings compared to 1 per 10,000 persons in high income countries [17, 18]. Lack of specialized mental health care workers continue to be a barrier to the delivery of mental health care in both specialized and primary care settings. Second, high levels of mental illness stigma (MIS) [19, 20] as well as poor mental health literacy (MHL) [21–23] that have been reported in SSA continue to hinder delivery of mental health care. Jorm (1997) [24] defines MHL as the ability to recognize the signs and symptoms of mental illnesses, know their causes, and identify sources of help (both lay and professional). The low level of MHL and high MIS are significant barriers to accessing care especially in light of the COVID-19 where there has been severe disruptions in movement of people to access care—in SSA settings, mental health care is mainly delivered at health facilities [25, 26]. Novel means of improving MHL and reducing MIS in light of the COVID-19 pandemic are urgently needed.

One of the interventions that can help enhance knowledge about mental health is psycho-education. The American Psychiatry Association [27] defines psycho-education as improvement in the knowledge in the subject areas that serve the goals of treatment and rehabilitation. However, findings about the efficacy of psycho-education are conflicting. Whereas some studies show that psycho-education as adjunct therapy leads better outcomes including better self-efficacy [28, 29], others studies have not documented benefits related to psycho-education [30–32]. Moreover, low levels of literacy [33–35] in SSA means that psycho-education interventions may be difficult to deliver. Novel means of delivering psycho-education materials in SSA are urgently needed.

For the proposed study, we will document baseline prevalence of MHL and CMD. To the best of our knowledge, this will be among the very first studies to examine the effectiveness of a psycho-education intervention in a community setting in the context of the COVID-19 pandemic.

### Objective

For the proposed study, we will determine effectiveness of a psycho-education intervention delivered by village health team (VHT) members.

### Study outcomes

Our primary outcome will be a mean change in MHL between the two arms. The secondary outcome will be associations between the MHL and demographic and CMD variables assessed by way of regression analysis.

## Methods

### Study design and setting

For the proposed study, we will use an open-label cluster-randomized trial of 24 randomly chosen villages (*n* = 12 intervention and *n* = 12 control) in Kampala (*n* = 15), Wakiso (*n* = 3), Mukono (*n* = 4), and Masaka (*n* = 2) districts. Intervention and control villages are located at least 20 km apart to avoid contamination

### Eligibility criteria

Individuals will be eligible for recruitment if they have been residing in the selected villages for a minimum of 6 months and do not intend to migrate or move/shift residence in the next month after being approached. Individuals will be eligible for recruitment if they are at least 18 years of age. All participants will be required to provide written informed consent. For those who can neither write nor read, the consent form will be read to them by trained research assistants (RA) and they will use a thumb print soaked in an inkpad to provide informed consent. The RA will be graduate level social scientists with experience in data collection.

### Study procedure

(i) Development of materials. We will engage with the mental health department at the ministry of health of Uganda to develop information and education communication (IEC) material—the aim of the IEC materials is to address the key barriers to mental health care access (low MHL and high MIS). The VHTs will distribute the IEC materials (described in the methods section) in the intervention villages. In the control villages, VHTs will distribute the ministry of health of Uganda leaflets that contain the COVID-19 prevention strategies.

(ii) Randomization and sampling: We shall send a list of all the villages in the districts (5250) to our statistician (Ronald Kiguba). We will then employ the multistage sampling approach to select 840 participants from 24 randomly selected villages located in the 362 parishes (5250 villages) that span the study districts. These villages will then be provided to the PI who will contact the VHTs in the said villages. Study villages will be selected using the probability proportional to size approach (based on number of parishes/villages per district). Thus, we shall select 15 villages from Kampala district (3297 villages), 4 villages from Mukono district (813 villages), 2 villages from Masaka district (436 villages), and 3 villages from Wakiso district (704 villages). The villages are at least 20 km apart to minimize contamination between the study arms (individuals interacting with each other during the intervention).

(iii) Participant identification: We shall engage the VHT and village Local area leaders who are conversant with the terrain of the villages to accompany the RA’s in data collection process in every 12th household in their catchment area till they accrue 38 participants in their village who express interest in participating in the study. The engagement of VHTs and village leaders will help us to accrue the said sample size within a stipulated time since these individuals are well conversant with the village. This figure is based on an average of about 4710 households per village. Thus, 4710/38 is approximately every 12th household. Individuals who express interest in the study will be given 2 consent forms which they will read and sign should they decide to participate; they will retain one copy. RA’s will read out the consent form to individuals who can neither read nor write and obtain informed consent (participants will use their thumb print soaked in an inkpad to consent). Consent forms will be translated into Luganda. The RA’s will then collect the data. The raw data will be stored for a minimum of 7 years as per Uganda National Council for science and technology recommendations. For those from whom data cannot be collected, we will conduct a phone interview. The VHT will get the participant’s telephone contact and provide it to the project administrator who will pass them to research assistants. Individuals who do not have phones will be asked to provide a phone number of a next of kin through which the interviews (with the individual) can be conducted—interviews could be conducted using the VHTs’ phone if participants express the need to do so.

(iv) Baseline data collection: At baseline, trained research assistants will collect demographic (age, gender, education level and place of residence) information and administer the Mental health Knowledge Schedule (MAKS) [36] to assess for MHL, Kesler-10 (K-10) [37] to measure PD, the Generalized Anxiety Disorder scale (GAD-7) [38] to assess for GAD, Patient Health Questionnaire-9 (PHQ-9) [39] to assess for MDD, Post-Traumatic Stress Disorder-Primary Care Screen (PTSD-PC) [40] for PTSD, and the Alcohol Smoking and Substance Involvement screening Test (ASSIST) [41] to assess for SUD. This data will be collected from participants in both arms. During the baseline data collection, the IEC materials will be distributed to participants in the intervention arm and the ministry of health (MOH) brochures to participants in the control arm.

(v) The intervention: VHTs will distribute the IEC materials to the 12 intervention villages described above after collection of baseline data and encourage participants to read these materials. In the 12 control villages, VHTs will distribute the MOH brochures that contain information about (a) the signs and symptoms of the COVID-19 and the preventive measures, (b) the fact that individuals may suffer from stress during the pandemic, (c) the sources from where they can get psychosocial help, and (d) the contact details of the research team should they need more information. The information contained in the ministry of health brochures are the current standard of care for community members. The IEC materials will be distributed to individuals who participated in the baseline surveys.

(vi) Mitigating the possibility of contamination between the study arms: Being an educational intervention, it is not possible to blind the participants in the control villages from receiving information meant for those in the intervention arm. Thus, there is a chance that participants in the control villages will access IEC materials distributed to those in the intervention arm, leading to contamination of our findings. To avoid contamination, we will do the following: (i) randomize villages so that they are at least 20 km apart (with the limited human movements during the COVID-19 pandemic, we anticipate that there will be little by way of people sharing the IEC materials), (ii) conduct the assessments within a week of distributing the materials to limit the chances that the information will be widely circulated, and (iii) ask participants in the control arms if they accessed the IEC materials meant for the control group—we will document the number of individuals who fall in this category. We have no control of other educational interventions that may be instituted during the trial period and circulated using the mass media. However, these interventions are usually sanctioned by the MOH—we are already working with the MOH and will know the nature and extent of these interventions if any. In the event that individuals are provided with similar information, then we will find out how many of them received it and acted on them. However, the trial period is quite short (7–10 days) and we believe that we can engage the MOH to implement these interventions after our intervention is complete.

(vii) Follow-up data collection: The same trained RA’s will collect follow-up data within a week after the VHTs have distributed IEC materials. The RA’s will administer the instruments administered at baseline (MAKS, K-10, GAD-7, PHQ-9, PTSD-PC, and ASSIST). The RA’s who collected the baseline data will be the same ones to collect the follow-up data. They would have set up appointments for the follow-up interview and will know the terrain well enough. The time duration between baseline and follow-up is a week. However, in some situations where the participant cannot be accessed within the week, we will give an allowance of up to 4 weeks within which to conduct the follow-up interviews—should the participant not be available within the said 4 weeks, then we will consider them as lost to follow-up.

(viii) Access to services. The leaflets distributed to participants from both arms will contain our study team phone numbers; participants can call and get help if need be. We will request that individuals without phones or those who may not have the money to call contact their VHTs (who are residents of the village) and request to make the calls using the VHT’s telephone. Research assistants will then assess the participants from CMD, and make appropriate decisions including referral to the nearest MOH psychosocial team members.

### Adverse event reporting during the interviews

We anticipate that there will be minimal adverse events that are directly related to the study—mainly psychological distress resulting from answering the questions during the survey or loss of private data. However, in the event that we realize any severe form of distress, or loss of privacy/confidentiality, then we will report it promptly reported to the Makerere University school of Medicine Ethics Research Committee (SOMREC) and Uganda National Council for Science and Technology (UNCST).

### Community tracing

In the event that a participant cannot be accessed for the second interview after the baseline one, then, we will ask the VHT to visit the physical location and find out the reason for non-response. The VHT will be provided with a Log to record this kind of information which could be loss of a phone and change in phone number of withdrawal from the study among other reasons. If the participant cannot be contacted on phone and physically (may have moved house), then we will consider this a loss to follow-up.

### Sample size and statistical considerations

#### Hypothesis

We hypothesize that (a) the MHL levels are low in our communities in comparison to those observed in high income countries, (b) a psycho-education intervention delivered by VHTs will lead to a 50% increase in MHL levels in the intervention arm compared to the control arm (that this difference will be clinically important), (c) more participants in the intervention arm will contact our study team compared to the control arm—and that this difference will be clinically important, and (d) it will be cost effective to implement the intervention.

- Sample size for the baseline prevalence of study outcomes. We will use the Leslie-Kish formular for calculating the sample size for cross sectional studies.

The formula states that *n* = Z^2^ p (1-p)/E^2.^ (i); n is the sample size, (ii) E is the standard error (5%), (c) - is the standard normal deviation of 1.96 corresponding to 95% confidence interval, (d) p is the proportion of participants, 50%. The prevalence was estimated at 50% (conservative estimate for outcomes or effects sizes with limited literature to refer to), in this case prevalence of poor MHL in the Ugandan community.

Substituting; *n* = 1.96^2^ X 0.5 X (1-0.5)/0.05^2^ *n* = 384. For the population survey, we will use a community correction effect of 2. Thus, the sample size for the community sample will be 384X2 = 764. We will add a 10% sample size to cater for incomplete data mainly due to failure to complete an interview. Thus, the total sample size will be 840 participants (420 in the control and 420 in the intervention arms).

(b) Sample size for the RCT: To determine the effectiveness of the psycho-education intervention, we have calculated the size of effects that our sample will be able to detect a clinically important difference in MHL level between the intervention and control arms. The nature of the educational intervention makes randomization by clusters (villages) more appropriate in comparison to randomization by individuals or household.

To estimate the number of villages required per arm for a clinically important effect size of 50%, we assume power of 80% at the 95% confidence level, mean MHL score of 14.14 and standard deviation (SD) of 2.19 [42], and cluster size of 35 participants and coefficient of variation of 0.25, which yields ~ 8 villages per trial arm. Therefore, 24 villages (both arms) will be required, thus including up to 840 participants (420 per trial arm). The sample size of 24 clusters with 35 participants in each cluster will provide us with enough power to detect statistically significance difference between the two arms.

#### Data collection and management

Data will be collected on paper and then entered into a centralized, web-based data management scheme using the open source software REDCap™. Once the data has been received, it will be stored and only accessible by study staff using a computer locked password. We will code all the data and delink names that can be used to identify participants.

### Statistical analysis

Objective 1: We will report frequencies and percentages of the study outcomes and their 95% confidence intervals. We will use regression analyses to determine associations between the primary outcome (MHL) and the factors that may be associated with it (demographics, CMD).

Objective 2: An intention-to-treat analysis will be conducted to compare the groups at baseline and within 4 weeks to assess the effects of the intervention on MHL. We will use a Student t test to examine this difference. The dependent variable (MHL) will be calculated as a continuous variable. Independent variables including presence of PD, MDD, GAD, PTSD, or SUD will be presented as continuous and categorical variables. Baseline characteristics of the intervention and control arms will be compared at the 5% level to assess if successful randomization was achieved. Data on potential confounders and effect modifiers, including variables that fail to achieve successful randomization (e.g., socio-demographic parameters) shall be used to control for confounding and effect modification. Between-subject analysis at week 4 will be used to assess the direct effect of the intervention by determining if there is a significant difference between the mean MHL scores in the intervention and control arms. Within-subject analysis will be performed among participants in the intervention arm by applying the Generalized Estimating Equations method on repeated measures data.

### Data safety management board

The data safety management board (DSMB) will be comprised of Prof Eugene Kinyanda (psychiatrist and senior epidemiologist), Dr. Moses Ocan (pharmacologist and IRB member), and Dr. Paul Bangirana (clinical psychologist with vast experience in conducting RCT) from Makerere University. The PI will have periodic meetings with the DSMB and provide them with reports about the study activities using an agreed template. There will be at least two meetings during the study period one at the beginning and another midway through the study. Members of the DSMB will make a visit at the Butabika Hospital where data will be stored. During this visit, the study procedures (SOPs and manuals), study responsibilities (delegation log), site facilities (data room), study materials (stationary and administrative materials), and the recruitment and retention plan will be discussed.

## Ethical considerations

A number of ethical considerations are worth considering.

### Potential risks

#### Psychological distress

Participants may feel distressed when talking about their illnesses during assessments. We will train our RA to be able to terminate the interviews in the event of severe psychological distress that results from the interview. That said, beyond the distress that will be experienced by participants, this will be a minimal risk study.

#### Confidentiality

To minimize the risk of loss of privacy and confidentiality, only the investigators will have access to the study records and test results and the link between personal identifying information and study data. No individual identities will be used in any reports or publications associated with the data from this study. All soft copies of the data will be stored in password locked computers. Hard copies of the questionnaires will be stored in locked file cabinets at the study offices in Butabika National Referral Hospital.

## Discussion

Findings from this research will guide policy and practice regarding the integration of mental health services in the community in the context of epidemic preparedness and response.

## Trial status

This is the final study protocol as of 08 November 2020. Recruitment will commence in early December 2020, and it is anticipated to get completed by January 2021

## Data Availability

Datasets will be available upon request from the investigators

## Abbreviations

CMD: Common mental disorders
MDD: Major depressive disorders
GAD: Generalized anxiety disorders
PTSD: Post-traumatic stress disorders
SUD: Substance misuse disorders
MHL: Mental health literacy
MIS: Mental illness stigma
VHT: Village health team (VHT)
MOH: Ministry of Health members
SOMREC: Makerere University school of Medicine Ethics Research Committee
UNCST: Uganda National Council for Science and Technology

## References

[1] Mazza C, Ricci E, Biondi S, Colasanti M, Ferracuti S, Napoli C (2020). A nationwide survey of psychological distress among Italian people during the COVID-19 pandemic: immediate psychological responses and associated factors. Int J Environ Res Public Health.

[2] Qiu J, Shen B, Zhao M, Wang Z, Xie B, Xu Y (2020). A nationwide survey of psychological distress among Chinese people in the COVID-19 epidemic: implications and policy recommendations. Gen Psychiatry.

[3] Zhang J, Lu H, Zeng H, Zhang S, Qifeng D, Jiang T (2020). The differential psychological distress of populations affected by the COVID-19 pandemic. Brain Behav Immun 2020.

[4] Substance Abuse and Mental Health Services Administration (SAMHSA) (2020). Warning signs and risk factors for emotional distress.

[5] Beaglehole B, Mulder RT, Frampton CM, Boden JM, Newton-Howes G, Bell CJ (2018). Psychological distress and psychiatric disorder after natural disasters: systematic review and meta-analysis. Br J Psychiatry..

[6] Jackson JL, Houston JS, Hanling SR, Terhaar KA, Yun JS (2001). Clinical predictors of mental disorders among medical outpatients. JAMA Intern Med.

[7] Manninen P, Heliovaara M, Riihimaki H, Makela P (1997). Does psychological distress predict disability?. Int J Epidemeol..

[8] Walker ER, McGee RE, Druss BG (2015). Mortality in mental disorders and global disease burden implications; a systematic review and meta-analysis. JAMA Psychiatry..

[9] Too LS, Spittal MJ, Bugeja L, Reifels L, Butterworth P, Pirkis J (2019). The association between mental disorders and suicide: a systematic review and meta-analysis of record linkage studies. J Affect Disord.

[10] Correll CU, Solmi M, Veronese N, Bortolato B, Rosson S, Santonastaso P, Thapa-Chhetri N, Fornaro M, Gallicchio D, Collantoni E, Pigato G, Favaro A, Monaco F, Kohler C, Vancampfort D, Ward PB, Gaughran F, Carvalho AF, Stubbs B (2017). Prevalence, incidence and mortality from cardiovascular disease in patients with pooled and specific severe mental illness: a large-scale meta-analysis of 3,211,768 patients and 113,383,368 controls. World Psychiatry..

[11] Whiteford HA, Ferrari AJ, Degenhardt L, Feigin V, Vos T (2015). The global burden of mental, neurological and substance use disorders: an analysis from the Global Burden of Disease Study 2010. PLoS One..

[12] Vigo DV, Kestel D, Pendakur K, Thornicroft G, Atun R (2019). Disease burden and government spending on mental, neurological, and substance use disorders, and self-harm: cross-sectional, ecological study of health system response in the Americas. Lancet Public Health..

[13] Lopes AP, Macedo TF, Coutinho ESF, Figueira I, Ventura PR (2014). Systematic review of the efficacy of cognitive-behavior therapy related treatments for victims of natural disasters: a worldwide problem. PLoS One.

[14] Brown RC, Witt A, Fegert JM, Keller F, Rassenhofer M, Plener PL (2017). Psychosocial interventions for children and adolescents after man-made and natural disasters: a meta-analysis and systematic review. Psychol Med.

[15] Hoskins M, Pearce J, Bethell A, Dankova L, Barbui C, Wietse A. Tol, et al. (2015). Pharmacotherapy for post-traumatic stress disorder: systematic review and meta-analysis. Br J Psychiatry..

[16] Sullivan GM, Neria Y (2009). Pharmacotherapy in post-traumatic stress disorder: evidence from randomized controlled trials. Curr Opin Investig Drugs..

[17] Burvill PW (2017). Looking beyond the 1:10,000 ratio of psychiatrists to population. Aust N Z J Psychiatry..

[18] Psychiatrists and nurses (per 100 000 population) [Internet]. 2017 [cited 09.August. 2017].

[19] Egbe CO, Brooke-Sumner C, Kathree T, Selohilwe O, Thornicroft G, Petersen I. Psychiatric stigma and discrimination in South Africa: perspectives from key stakeholders. BMC Psychiatry. 2014;14(191) 10.1186/471-244X-14-191. 10.1186/1471-244X-14-191 PMC4099203 24996420

[20] Kapungwe A, Cooper S, Mwanza J, Mwape L, Sikwese A, Kakuma R (2010). Afr J Psychiatry (Johannesbg). Mental Illn Stigma Discrimination Zambia.

[21] Okello ES, Ekblad S (2006). Lay concepts of depression among the Baganda of Uganda: a pilot study. Transcult Psychiatry.

[22] Hailemariam KW (2015). Perceived causes of mental illness and treatment seeking behaviors among people with mental health problems in Gebremenfes Kidus Holy Water Site. Am J Appl Psychol.

[23] Teferra S, Shibre T. Perceived causes of severe mental disturbance and preferred interventions by the Borana semi-nomadic population in southern Ethiopia: a qualitative study. BMC Psychiatry. 2012;12(79) 10.1186/471-244X-12-79. 10.1186/1471-244X-12-79 PMC3416742 22789076

[24] Jorm AF (2000). Mental Health Literacy. Public belief about mental disorders. Br J Psychiatry.

[25] Kigozi F, Ssebunnya J, Kizza D, Cooper S, Ndyanabangi S, The Mental Health and Poverty Project. An overview of Uganda's mental health care system: results from an assessment using the world health organization's assessment instrument for mental health systems (WHO-AIMS). Int J Ment Health Syst. 2010;4(1). 10.1186/752-4458-4-1. 10.1186/1752-4458-4-1 PMC2831025 20180979

[26] Docrat S, Besada D, Cleary S, Daviaud E, Lund C (2019). Mental health system costs, resources and constraints in South Africa: a national survey. Health Policy Plan.

[27] Baum J, Frobose T, Kraemer S, Rentrop M, Pitschel-Walz G (2006). Psychoeducation: a basic psychotherapeutic intervention for patients with schizophrenia and their families. Schizophr Bull.

[28] Shorey S, Chan SW, Chong YS, He HG (2015). A randomized controlled trial of the effectiveness of a postnatal psychoeducation programme on self-efficacy, social support and postnatal depression among primiparas. J Adv Nurs.

[29] Elliott SA, Leverton TJ, Sanjack M, Turner H, Cowmeadow P, Hopkins J (2000). Promoting mental health after childbirth: a controlled trial of primary prevention of postnatal depression. Br J Clin Psychol..

[30] Hayes BA, Muller R, Bradley BS (2001). Perinatal depression: a randomized controlled trial of an antenatal education intervention for primiparas. Birth..

[31] Alimonos LA, Simpkins G, DeAngelo M, Chernoff S, Hunter K, Khandelwal M. Feasibility of psychoeducational sessions in pregnant women at risk for postpartum depression: a prospective study. Obstet Gynecol. 2015. 10.1097/01.AOG.0000462764.03433.fa.

[32] Stamp GE, Williams AS, Crowther CA (1995). Evaluation of antenatal and postnatal support to overcome postnatal depression: a randomized, controlled trial. Birth..

[33] Ganasen KA, Parker S, Hugo CJ, Stein DJ, Emsley RA, Seedat S (2008). Mental health literacy: focus on developing countries. Afr J Psychiatry (Johannesbg)..

[34] Atilola O (2015). Level of community mental health literacy in sub-Saharan Africa: current studies are limited in number, scope, spread, and cognizance of cultural nuances. Nord J Psychiatry.

[35] UNESCO Institute for Statistics (2015). Adult and youth literacy.

[36] Evans-Lacko S, Little K, Meltzer H, Rose D, Rhydderch D, Henderson C, Thornicroft G (2010). Development and psychometric properties of the mental health knowledge schedule. Can J Psychiatry..

[37] Kessler RC, Andrews G, Colpe LJ, Hiripi E, Mroczek DK, Normand SLT (2002). Short Screening scales to monitor population prevalences and trends in non-specific psychological distress. Psychol Med..

[38] Spitzer RL, Kroenke K, Williams JBW, Löwe B (2006). A brief measure for assessing generalized anxiety disorder: the GAD-7. Arch Intern Med.

[39] Kroenke K, Spitzer RL, Williams JB (2001). The PHQ-9: validity of a brief depression severity measure. J Gen Intern Med.

[40] Prins A, Bovin MJ, Smolenski DJ, Marx BP, Kimerling R, Michael A. Jenkins-Guarnieri, et al. (2016). The Primary Care PTSD Screen for DSM-5 (PC-PTSD-5): development and evaluation within a veteran primary care sample. J Gen Intern Med.

[41] The Alcohol, Smoking and Substance Involvement Screening Test (ASSIST): manual for use in primary care. [Internet]. World Health Organization 2010.

[42] Kutcher S, Wei Y, Gilberds H, Ubuguyu O, Njau T, Brown A, et al. A school mental health literacy curriculum resource training approach: effects on Tanzanian teachers’ mental health knowledge, stigma and help-seeking efficacy. Int J Ment Health Syst. 2016;10(50). 10.1186/s13033-016-0082-6. 10.1186/s13033-016-0082-6 PMC4973111 27493684

